# Pupillometry: A Simple and Automatic Way to Explore Implicit Cognitive Processing

**DOI:** 10.21769/BioProtoc.5265

**Published:** 2025-04-05

**Authors:** Tian Yuan, Li Wang, Yi Jiang

**Affiliations:** 1 School of Psychology, Shanghai Normal University, Shanghai, China; 2 State Key Laboratory of Cognitive Science and Mental Health, Institute of Psychology, Chinese Academy of Sciences, Beijing, China; 3 Department of Psychology, University of Chinese Academy of Sciences, Beijing, China

**Keywords:** Pupil size, Emotion perception, Biological motion, Local motion, Non-biological motion, Autistic traits

## Abstract

Pupil size is a non-invasive and highly sensitive technique used to measure changes in pupil diameter. It not only responds to light but also reflects inner cognitive processes (e.g., attention and emotion perception). Recently, it has been introduced to the traditional cognitive neuroscience field as a useful tool to objectively and sensitively capture the current cognitive state and its temporal dynamics. Importantly, this index is automatic and requires no explicit reports, thus it could be used to investigate the rarely explored realm of implicit cognitive processing. Here, we describe a comprehensive protocol that records pupil responses during the passive viewing of emotional biological motion (BM). Our results reliably reveal the multi-level implicit processing mechanism of BM emotion, as indicated by the fine-grained emotion processing in intact BM and the rapid but rather coarse emotion processing in local BM. Moreover, the emotion modulation effects observed in intact BM are indicative of individual autistic tendencies. We believe this protocol could be adapted to unveil the automatic processing of emotions and other attributes in social signals and further assist the early detection of social-cognitive disorders (e.g., autism).

Key features

• Pupil measurements could automatically and objectively reflect current cognitive states, which is useful for unfolding the implicit cognitive processes and related time courses.

• It requires no explicit reports; thus, with modifications, this procedure could be utilized in a wide range of populations (e.g., infants, patients) and even animals.

• Autistic traits strongly correlate with observed emotion-related pupil responses, revealing the potential application of pupil measurements in the detection of social cognitive deficits.

• This protocol has been found to reveal the multi-level emotion processing of biological motion, which is further validated by a test-retest replication.

## Graphical overview


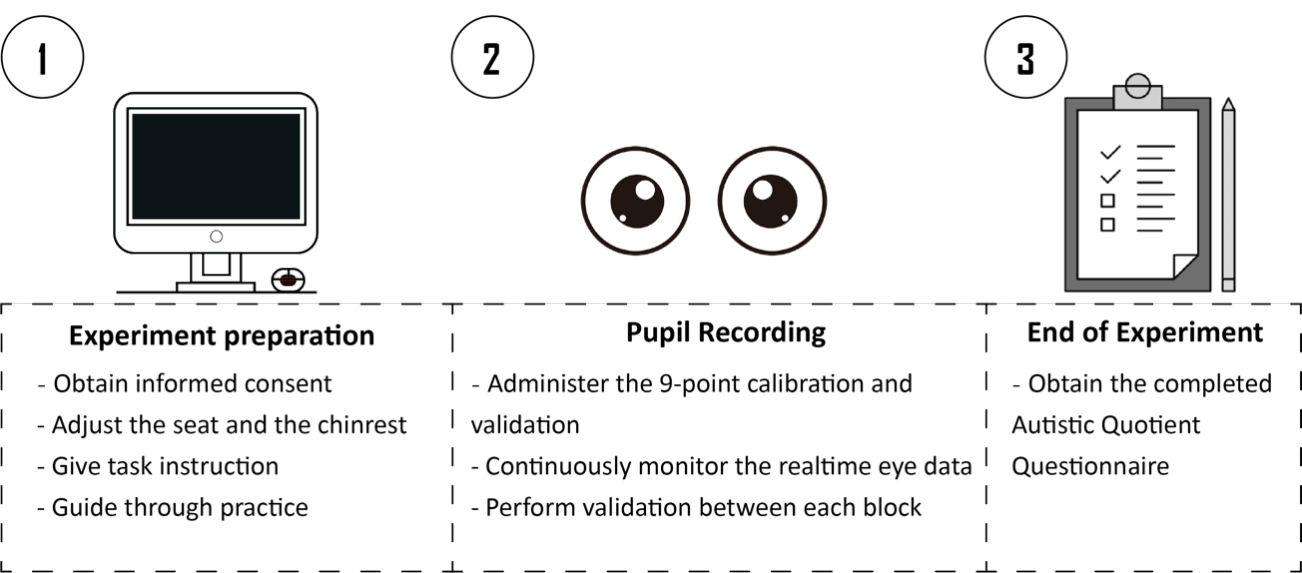


## Background

Pupil size refers to the diameter of the spherical opening in the eye, which is regulated by the relative activation of the sympathetic (pupil dilation) and parasympathetic (pupil constriction) branches of the automatic nervous system [1]. It is primarily mediated by the locus coeruleus norepinephrine (LC-NE) and could reflect the activity of related subcortical nuclei [2]. Importantly, pupil size not only responds to the strength of external physical light but also reflects the underlying cognitive state [3]. Moreover, it spontaneously captures the current cognitive state and is thus useful for revealing the time course of the related cognitive processing [4–7]. Recently, this index was introduced to the cognitive neuroscience field as a powerful tool to unveil the processing mechanism of multiple cognitive functions in healthy adults [3], infants [8,9], patients (e.g., ASD) [10,11], and even animals [12,13], thus offering a reliable psychophysiological measurement that applies to diverse populations and species.

Perceiving emotions from diverse social signals is a fundamental cognitive ability that ensures effective communication and social functioning [14]. Facial expressions present the most common non-verbal social signals conveying others’ affective states [15]. In addition to faces, the movement of biological organisms, portrayed by several point-light dots attached to the major joints [16,17], could also convey significant emotional information [18]. Existing studies have mostly investigated the emotion processing of BM using the active emotion detection or recognition paradigm [19–21]. Nonetheless, the encoding of emotional information also involves a rather automatic and implicit process that is independent of the participant’s explicit identifications [22–25]. Moreover, this implicit aspect of emotion processing could be even more effective in identifying individual differences and social deficits [26–28], as it requires the intuitive processing of emotions that could not be learned. Notably, pupil response, which requires no active reports and adds no extra task requirements to the cognitive process, potentially serves as a promising approach to reveal this implicit emotion perception process [29].

Here, we describe a detailed protocol that implements the pupil recording technique together with the passive viewing paradigm to unveil the automatic emotion processing of biological motion (BM). In addition, individual autistic scores are also collected using the autistic quotient (AQ) questionnaire in order to examine the potential application of this protocol in identifying autistic tendencies [30]. This protocol has been tested in intact, inverted, local, and non-biological motion sequences, and was found to uncover a multi-level processing mechanism of BM emotions, as indicated by a fine-grained but late emotion processing in intact BM and a coarse but rapid emotion processing in local BM. Besides, the emotion-related pupil modulation effects were indicative of individual autistic tendencies, which was further validated in a test-retest replication experiment. We expect this protocol to be informative in revealing the automatic emotion processing of various social signals. More importantly, given that this protocol requires no explicit reports, it also has the potential to be utilized in the detection of social cognitive disorders/tendencies in a wide range of populations with modifications.

## Materials and reagents

**Figure 1.**
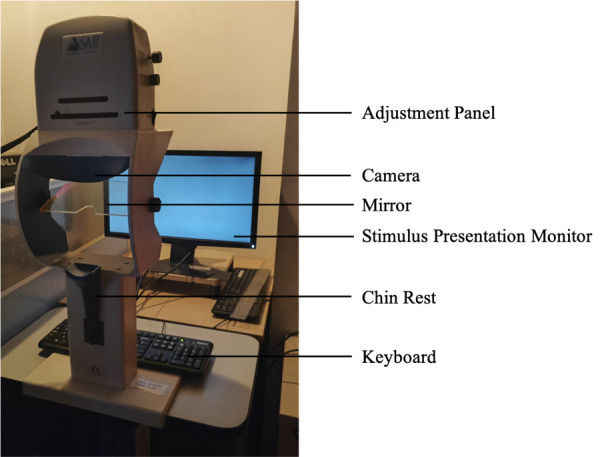
Experiment setup in the subject area. The eye-tracking system is comprised of a stimulus presentation monitor, a keyboard, and an eye tracker.

1. Setup for subjects’ seating area (Figure 1):

a. Table for stimulus presentation monitor and chin rest.

b. Stable chair for the subject to sit.

2. Personal items for cleaning the equipment after the experiment, like tissue paper, alcohol pad, etc.

3. Online link to the Autistic Quotient Questionnaire [30].

## Equipment

1. SMI iView X High-Speed 500 eye-tracking system; includes the infrared eye-tracking camera, the chin rest, and the eye data acquisition computer (System ID: IVS-X-1104-05510-FST)

2. Stimulus presentation monitor (in subject area): Alienware 2310 (LED, 1,920 × 1,080 pixels, refresh rate: 60 Hz)

3. Stimulus presentation computer (Dell Precision T3500)

4. Eye-data presentation monitor (Dell 2209WA)

## Software and datasets

1. Stimulus presentation: custom code built using MATLAB 2016b (MathWorks, Inc.) with the Psychtoolbox extensions [31,32] (https://github.com/Psychtoolbox-3/Psychtoolbox-3/tree/3.0.20.3)

2. Eye recording: SMI iView X Hi-Speed software (SensoMotoric Instruments, Berlin, Germany; System ID: IVS-X-1104-05510-FST)

3. Data analysis:

a. MATLAB 2016b, with the following toolboxes/code:

i. Cluster-based permutation analysis function (clust_perm1.m) from Mass_Univariate_ERP_Toolbox [33] (https://github.com/dmgroppe/Mass_Univariate_ERP_Toolbox)

ii. gramm toolbox. Version 3.0.0 is used in the current protocol. Newer versions of the software could also be adopted if this version is not available

iii. Custom code for preprocessing, group analysis, and visualization (https://osf.io/qe39w/?view_only=06fab1bb32794ecdb3eabb2f56ea7d6d)

b. Jamovi. Version 2.3.28 is used in the current protocol. Newer versions of the software could also be adopted if this version is not available (https://www.jamovi.org/) [34]

c. GPower. Version 3.1.9.7 is used in the current protocol. Newer versions of the software could also be adopted if this version is not available (https://www.psychologie.hhu.de/arbeitsgruppen/allgemeine-psychologie-und-arbeitspsychologie/gpower) [35]

## Procedure

In the following sections, we describe in detail the procedure for experiment preparation, eye recording, and data analysis used in Yuan et al. [11].

**A. Participant enrollment and preparation**

1. Recruit participants. In our case, participants were recruited on a voluntary basis through advertisements and social media. The targeted sample size can be calculated using G*Power according to the experimental design and expected statistical power [35].

a. Participants should have normal or corrected-to-normal vision. Individuals with astigmatism exceeding 100 degrees, strabismus, color blindness, color weakness, or serious ophthalmic diseases should be screened out from the experiment. Additionally, participants who are taking medications that could influence pupil response should also be excluded. Those with myopia are required to wear standard transparent optical glasses that correct their vision without any color filtering (especially not blue-light blocking glasses, photochromic glasses, sunglasses, etc.). The experimenter should implement comprehensive pre-experiment screening to identify any visual impairments that might affect pupil recording.

b. Eligible participants were enrolled in the experiment and provided with pre-experiment instructions to ensure the quality of eye-tracking data. Specifically, they should obtain adequate rest and limit the consumption of stimulants (e.g., caffeine, alcohol) 24 h preceding the experiment. Additionally, participants were instructed not to wear eyelashes or makeup on the day of the experiment to minimize potential interference with the collection of accurate eye data.

**B. Experiment room setup**

**Figure 2.**
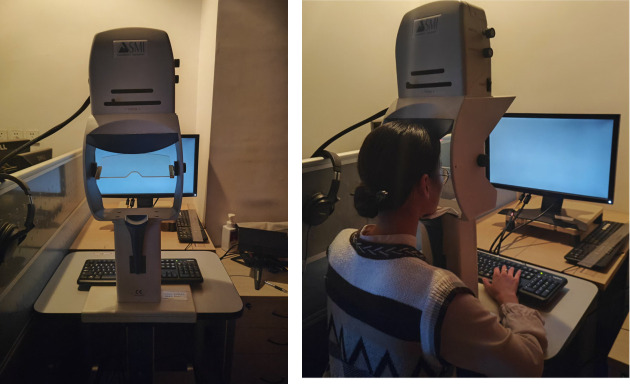
SMI eye-tracking system and a subject performing the task. The stimulation presentation monitor is positioned 60 cm in front of, and aligned with, the eye tracker to facilitate easy fixation at the center of the screen for the participants. The participants should be seated in a comfortable position, with their heads supported by the chinrest. They are asked to maintain fixation at the center of the screen and not to blink during the stimulus presentation.

1. The experiment room should be comprised of a stimulus presentation computer, an eye-data acquisition computer, and the SMI eye-tracking system, and be dimly lit to match the display’s brightness.

2. Tell subjects to comfortably sit on a stable chair and place their chin on the chinrest of the eye-tracking machine. Their head should be supported by cheek and headrests mounted on the eye tracker.

3. Place the monitor 60 cm in front of and at the level of the eye tracker to make sure that participants can easily place their fixations at the center of the screen. In our case, the stimuli presentation monitor subtended a width of 51 cm (48.92°) and height of 28.4 cm (27.09°). The chin rest or its components never obstructed the monitor, and the entire monitor was always visible to the subjects (see Figure 2).

**C. Experiment preparation**

1. On the arrival of the subject, brief the participant about the experiment procedure and obtain the written consent before starting. The consent form should provide a brief overview of the entire experiment, including the purpose, procedure, risks, voluntary participation, confidentiality, and compensation.

2. Participants must be informed that they need to remain as still as possible to minimize head movements, and that scheduled breaks are given during the experiment for them to relax.

3. For participants having difficulty controlling their head movements (e.g., infants, children), it is recommended to adopt eye recording equipment that utilizes stickers or markers as reference points to help track the individual’s head position (e.g., Eyelink).

4. For animal studies, specialized equipment and techniques are required to ensure accurate eye-tracking data with limited participant cooperation.

5. Ask the participant to sit on the subject’s chair and place their chin on the chin rest.

6. Assist the participant in adjusting the height of the chin rest or the chair to ensure the participant is sitting in a comfortable position.

7. Present detailed experiment instructions on the screen, as follows:

“Welcome to the experiment! This experiment is an eye-recording experiment. During the experiment, a central fixation cross will first appear on the screen. After that, a point-light motion sequence will appear at the center of the screen. Please maintain your attention on the central fixation cross and try not to blink during the stimulus presentation. After the stimuli disappear, you are allowed to take a break and can proceed to the next trial by pressing the “space” key. The experiment will last for approximately 30 min, with fixed breaks in between. During the break, you may close your eyes to rest, but please do not move your head. If you have any questions, please consult the experimenter. When you are ready, press the spacebar to begin the practice.”

8. Make sure the participant fully understands the instructions and has no further doubts.

9. Lead the participant through a practice session of about five trials to familiarize with the task. Extended practice sessions are available in case the participant fails to comprehend the procedure.

10. For participants having trouble reading, it is recommended to present this introduction orally, supplemented with visual aids. Moreover, the practice section could be used as a vivid demonstration of the experimental procedure. Note that the experiment should not start the experiment before making sure that the participant has no further doubts.

11. Set the eye-tracker at a sampling rate of 500 Hz (a higher sampling rate is available for more detailed analysis) and record only the left eye data. This choice is a common practice in previous studies, but you may also record the right eye or both eyes [36]. Note that for the analysis of microsaccades, it is recommended to record both eyes’ data as those are better defined by binocular eye movements [37].

**D. Stimuli**

1. Use intact BM, inverted BM, non-biological motion, and local BM sequences in each of the four experiments.

2. Take the original BM stimuli from the stimuli set developed by Troje [17,38]. In this set, each BM comprises 15 point-light dots depicting the motions of the head, pelvis, thorax, and major joints (i.e., shoulders, elbows, wrists, hips, knees, and ankles). The emotional state is indexed by a normalized Z score on an axis that reflects the differences between happy and sad walkers in terms of a linear classifier. The scores are computed within a 10-dimensional sub-space spanned by the first 10 principal components based on a Fourier-based representation of observers’ emotional ratings of 80 actual walkers (half male).

3. Adopt the BM walker that scored 0 on the linear axis as the neutral BM, the walker 6 SDs into the happy part of the axis as the happy walker, and the walker 6 SDs into the sad part of the axis as the sad walker (see https://www.biomotionlab.ca/html5-bml-walker/ for an interactive animation).

4. Create the intact BM by turning the BM walkers 45 degrees leftward or rightward to maximize the visibility of expressive bodily cues [39]. Thus, the emotional (happy, sad, or neutral) BM walkers with two walking directions (45° leftwards or rightwards) are used.

5. Create the inverted version of BM stimuli by mirror-flipping the upright BM vertically to control for the confound of low-level visual features, as the inverted BM stimuli share the exact perceptual features with their upright counterparts.

6. Develop the non-biological motion stimuli through acceleration elimination to investigate the role of local motion features in the emotion processing of BM. In other words, make each dot move along the original path with a constant speed equal to the average speed of the dot [40]. Such manipulation disrupts the local motion feature of BM stimuli but keeps the trajectories of individual point lights unchanged.

7. Develop the local BM stimuli by preserving only the local motion feature to explore its role in emotion processing. More specifically, create the local BM by randomly relocating the point-light dots within the region occupied by the original BM walker. In this manner, the local motion feature is preserved while the global configuration of the BM stimulus is entirely disrupted [41] (see Video 1 for stimuli demonstration).

**Video 1.**
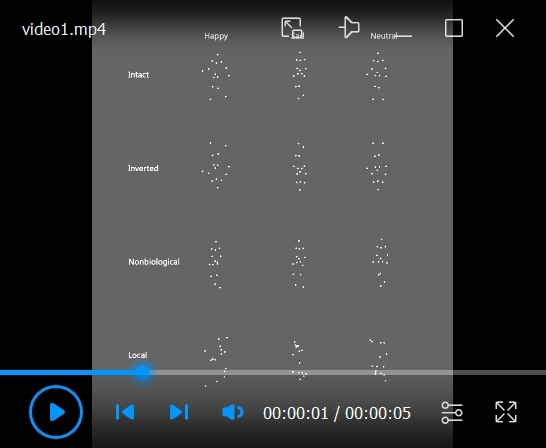
Video demonstrating the happy/sad/neutral intact, inverted, non-biological, and local motion sequences. The intact happy/sad/neutral biological motion (BM) was taken from Troje. The inverted BM was created by mirror-flipping the intact BM. The non-biological motion was created through the removal of acceleration. The local BM was generated by randomly relocating the point-light dots within the region occupied by the original BM walker.

**E. Pupil recording**

**Figure 3.**
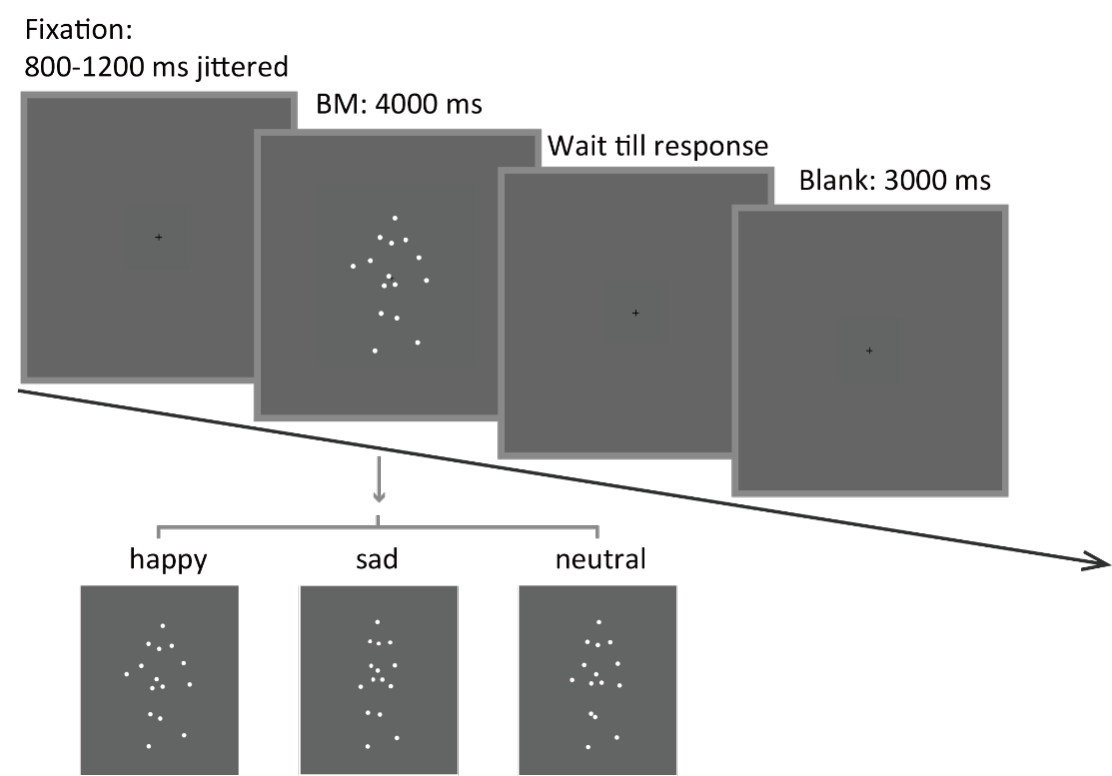
Schematic representation of the experimental procedure and the stimuli. A happy/sad/neutral biological motion (BM) walker turning 45° leftwards or rightwards was presented at the center of the screen for 4,000 ms. Participants were instructed to fixate on the BM stimuli during stimulus presentation and to continue the procedure through key pressing.

1. Before the recording of the formal experiment, lead the participant through a nine-point calibration procedure to calibrate the eye tracker.

2. Run the custom MATLAB script to present the participant with the point-light BM stimuli.

3. Each trial begins with a central fixation cross (0.2°× 0.2°) with variable duration (800–1,200 ms). This is designed to obtain a reliable baseline pupil size. Then, an upright happy/sad/neutral point-light walker (half leftwards and half rightwards) is presented centrally for 4,000 ms. Ask the participants to fixate at the center of the screen and passively view the BM stimulus without blinking. After the stimulus disappears, the participant can continue the procedure by pressing the space bar. Next, a blank screen is displayed for 3,000 ms to allow the pupil size to return to normal (Figure 3).

4. The experiment consists of four blocks, each comprising 30 trials, and participants are given a short break after every block. Before the start of each block, administer a validation procedure of the eye-tracker to ensure the quality of the recorded data. Participants are asked not to move their heads within each block.

5. In the event of an accidental head movement, re-administer the calibration procedure to confirm that the participant's gaze remains accurately aligned with the center of the screen.

6. During the recording, continuously monitor the real-time eye data to ensure the following: (1) the participant actively maintains their fixation at the center of the screen; (2) the participant is not blinking excessively; (3) the eye recorder precisely captures the position and the size of the pupil.

7. After each block, it is encouraged to check in with the participant regarding their current state and make sure the participant takes a full break before continuing the procedure.

8. At the end of all experiments, participants are required to complete the 50-point Autism-spectrum Questionnaire (AQ), which measures the degree of autistic traits in the normal population (Baron-Cohen et al. [30]).

## Data analysis

Data analysis was conducted using mainly the custom MATLAB scripts; thus, basic knowledge of MATLAB is required while performing data analysis. Nevertheless, we have attempted to describe the detailed data analysis procedure to enable future researchers to easily transfer the whole procedure to other data analysis software such as R or Python. Note that many R-based and Python-based toolboxes are available to perform similar analyses [42].

**A. Data preparation**

Use the IDF Converter 3.0.16 software to convert the original file from .idf format to a more universally accessible .txt format. Import eye data into MATLAB 2016b for further analysis. Specifically, the time series, the event marker, the width and length of the pupil, the x and y position of gaze, and the corneal reflection are extracted from the raw data. The extracted data are subsequently segmented into epochs, spanning from -0.4 s before stimulus onset to 0.1 s following the offset, based on the time series and markers.

**B. Preprocessing**

Screen the extracted epoch for each trial to check the overall data quality. Typically, a good trial should show few blinks, no missing data, and no sudden shifts of gaze positions. To identify abnormal trials, it is recommended to visualize the related indexes, i.e., pupil size, gaze position, and corneal reflection, at each time point within each trial for clearer and better data inspection. The width and length of the pupil both reflect the pupil size, and they are highly correlated. Given that the pupil response evoked by cognitive processing is mild (tenths of a millimeter), the line representing the pupil width and length should be continuous and relatively stable. The gaze positions (x position, y position) reflect where the gaze is placed during stimuli presentation, which should also show little fluctuations. The corneal reflection refers to the reflection of light off the surface of the cornea (i.e., the bright part of the eye). A relatively small corneal reflection would indicate a constant tracking of the eye.

**Figure 4.**
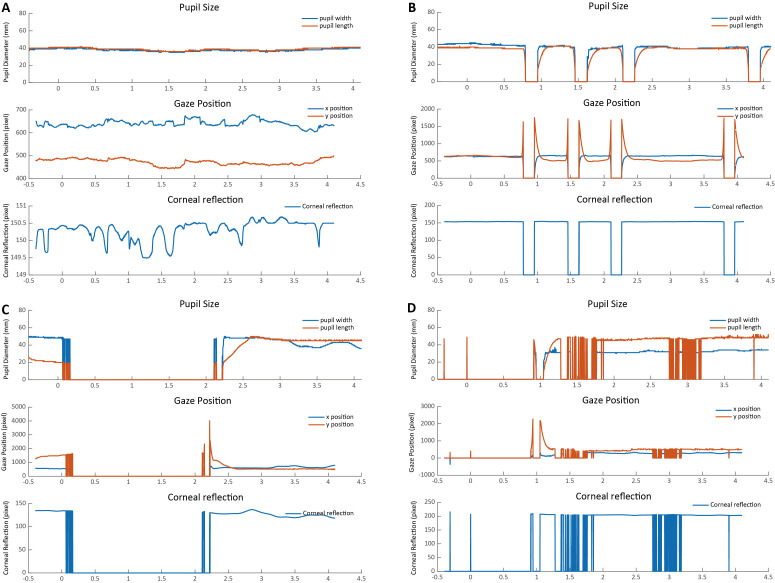
Examples of good and bad trials. (A) The pupil size, gaze position, and corneal reflection in a typical good trial should be continuous and stable. (B) A typical bad trial with too many eye blinks. (C) A typical bad trial with large missing data. (D) A bad trial with large noise.

Overall, the graph of a good trial should be depicted by continuous horizontal lines in pupil size, gaze positions, and corneal reflection with no extreme values (see Figure 4A). Instead, a sudden drop/increase in pupil size, gaze position, and corneal reflection that shortly returns to the normal level usually indicates a blink (Figure 4B). In addition, a long-lasting period of zero values or dramatic fluctuations usually indicate the loss of tracking of the eye (Figure 4C and D). Trials with too many blinks, missing data, and large variability in pupil sizes, gaze positions, and corneal reflection should be marked as bad trials and should be trimmed out from the follow-up analysis.

**Figure 5.**
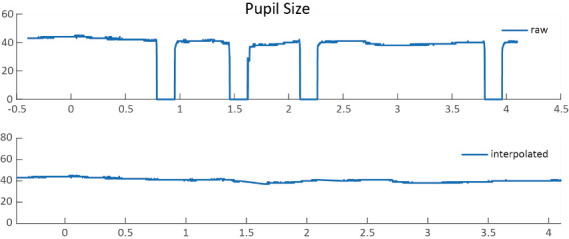
Raw data and interpolated data. The interpolated data were generated by averaging the values from the nearby time points (within a 50 ms time window) around the abnormal values.

During the bad-trial rejection procedure, it is suggested to retain the majority of the trials for statistical power. Trials with occasional blinks, artifacts, and gaze shifts should not be discarded, as they could be removed through interpolation. Specifically, pupil diameters that fall below 10 arbitrary units (a.u.), exceed 70 a.u., or deviate by 3 standard deviations from the mean, and gaze positions 3 standard deviations above or below the mean are identified. The linear interpolation is then applied to these marked abnormal values by averaging the values from the nearby time points (within a 50 ms time window) around the abnormal values to remove residuals (see Figure 5 for an example). Because this protocol is designed for the purpose of pupil diameter analysis, only abnormal pupil size values in each trial were interpolated.

**C. Group analysis**

The preprocessed epochs are grouped into different emotional conditions (sad, happy, neutral) according to the stimuli property. The pupil size data is then down-sampled to 20 Hz and baseline-corrected for each trial by subtracting the mean pupil size during the 200 ms pre-stimulus period.

**Figure 6.**
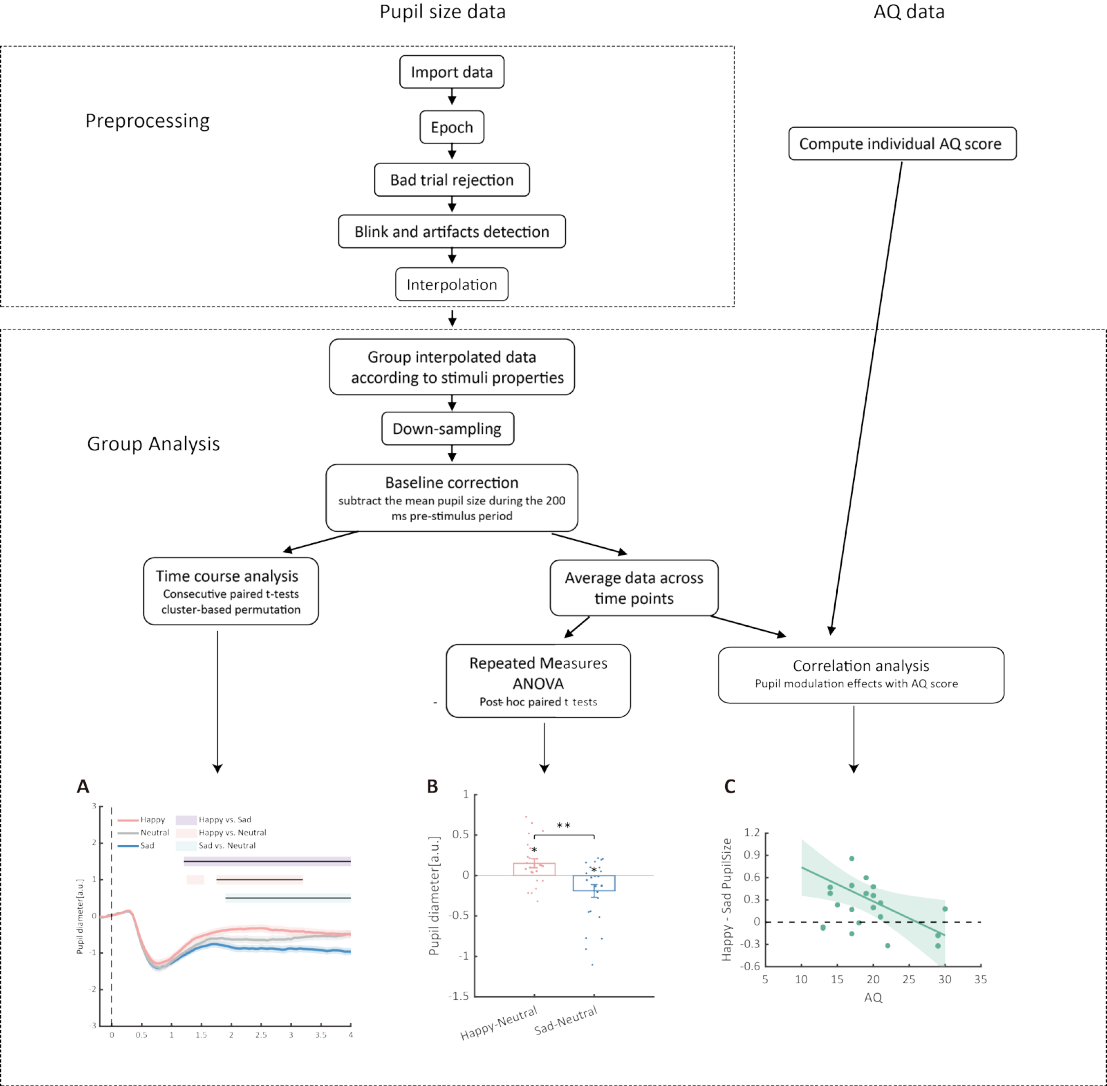
Detailed data analysis procedures. The raw pupil size data first underwent a preprocessing procedure, including epoching, bad trial rejection, blink and artifact detection, and interpolation. Subsequently, data were grouped according to the properties of the stimuli and downsampled to 20 Hz, followed by baseline correction based on the 200 ms pre-stimulus period. (A) Consecutive paired t-tests were performed on the preprocessed data to explore the time-course of pupil modulation effects. Solid lines represent pupil diameter under each emotional condition as a function of time (happy: red; sad: blue; neutral: gray); shaded areas represent the SEM between participants; colored horizontal lines indicate periods during which there are statistically significant differences among conditions at *p* < 0.05; and black horizontal lines indicate significant differences after cluster-based permutation correction. All pupil data are in arbitrary units (a.u.). (B) Additionally, a repeated measures ANOVA on the averaged pupil sizes was performed. Each point represents one individual data point. Error bars show standard errors of the mean. * *p* < 0.05, ** *p* < 0.01. (C) Finally, correlation analyses between averaged pupil sizes and Autistic Quotient (AQ) scores were conducted. The line and shaded areas denote a general linear model fit with a 95% confidence interval.

To depict the time course of pupil responses toward emotional BM, a consecutive paired-sample *t*-test across all time points comparing different emotional conditions was conducted. The cluster-based permutation analysis was applied to avoid potential problems brought about by multiple comparisons [43]. In this analysis, the computed t-values neighboring in time that exceeded a threshold (*p* < 0.05) were defined as clusters and then summed to produce a cluster mass. The cluster mass was compared with a null distribution, which was generated by 2,000 random permutations of the pupil data from different conditions. If the cluster mass fell beyond 95% of the null distribution (*α* = 0.05), it was deemed to be statistically significant. The pupil size was analyzed and reported in arbitrary units (a.u.) without transforming into the actual unit (mm), as the relative change of the pupil size was of main interest. Results of the time-course analysis are plotted for better demonstration: the raw data is smoothed using the nearby 50 time points; the colored horizontal lines indicate periods during which there are statistically significant differences among conditions at *p* < 0.05; and black horizontal lines indicate significant differences after cluster-based permutation correction (see Figure 6A).

Moreover, this protocol also computed the average pupil size for each emotional condition (happy, sad, or neutral) obtained by collapsing the pupillometry across all time points. Then, a one-way repeated measures ANOVA was conducted on the mean pupil size for each emotional condition (happy, sad, neutral) using the Jamovi software (version 2.3.28) [11]. If a main effect of emotional condition was observed, it would indicate that BM emotions significantly modulate pupillary responses. The post hoc paired-sample analysis (Bonferroni-corrected) was subsequently applied to directly compare pupil sizes under different emotional conditions (happy, sad, neutral) (see Figure 6B).

**Figure 7.**
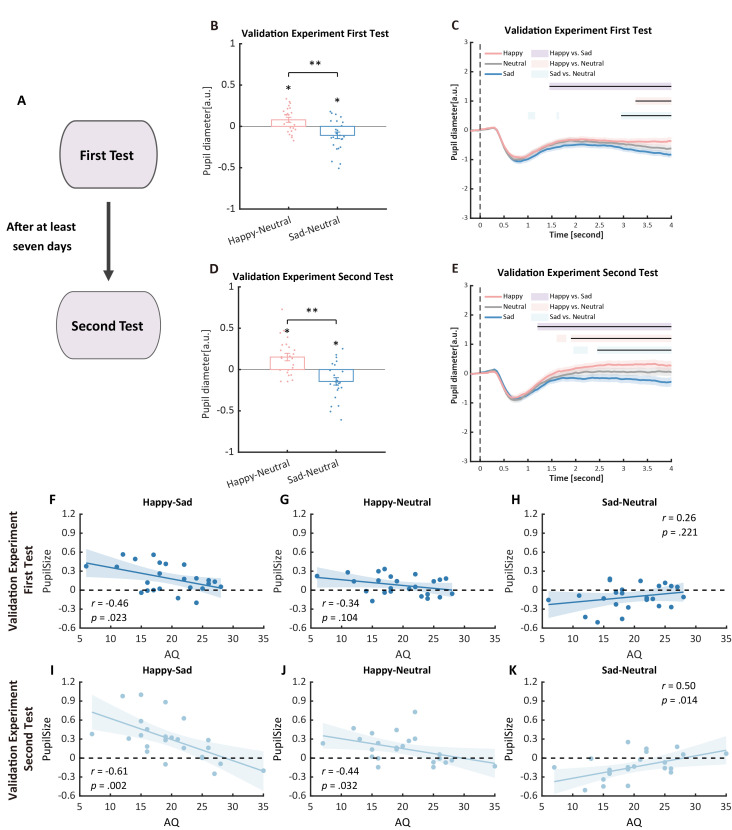
Procedure and results of the validation experiment. (A) Procedure of the validation experiment: participants were recruited to perform the pupil size task and were asked to return for a retest after at least seven days. (B, C) In the first test of the validation experiment, we successfully replicated the pupil modulation effects: the happy biological motion (BM) evoked a larger pupil response as compared to the sad and neutral BM, and the sad BM evoked a smaller pupil size than the neutral BM. (D, E) Such results were similarly observed in the retest. (F) Correlation results for pupil modulation effects and Autistic Quotient (AQ) scores. We replicated the negative correlation between the happy vs. sad pupil dilation effect and AQ. (G, H) No other significant correlations were found. (I) In the retest, the negative correlation between the happy vs. sad pupil dilation effect and AQ was similarly observed and even stronger. (J, K) The happy vs. neutral pupil dilation effect and the sad vs. neutral pupil constriction effect correlated with AQ in the second test.

The happy vs. sad pupil modulation indexes were subsequently computed by subtracting the average pupil size obtained in the happy condition from that obtained from the sad condition. The happy vs. neutral and sad vs. neutral pupil modulation effects were similarly computed to depict exact emotion modulation directions. These modulation effects were then correlated with individual autistic traits obtained using the Autistic Quotient (AQ) questionnaire. The gramm toolbox (version 3.0.0) was used to visualize the correlations (Figure 6C; see Figure 7 for a detailed demonstration of the data analysis procedure).

## Validation of protocol

This protocol has been validated with a test-retest procedure to assess the reliability of pupil sizes and AQ measurements. Specifically, a new group of 24 participants were recruited to perform an identical experiment procedure. Then, after at least seven days, they were asked to return to the lab for a retest. Previous pupil modulation effects and their correlations with autistic traits were successfully replicated in the first test and further validated in the second test. Moreover, in the second test, the correlation between pupil modulation effects and AQ became more significant (Figure 7, see also https://osf.io/qe39w/?view_only=06fab1bb32794ecdb3eabb2f56ea7d6d for the validation data).

This protocol or part of it has been used and validated in the following research article(s):

• Yuan et al. [11]. Multi-level processing of emotions in life motion signals revealed through pupil responses. *eLife.* 12: RP89873 (Figures 3 and 4).

• Cheng et al. [44]. Eye pupil signals life motion perception. *Attention, Perception, & Psychophysics.* 86(2): 579–586 (Figure 2).

• Cheng et al. [45]. The eyes have it: Perception of social interaction unfolds through pupil dilation. *Neuroscience Bulletin*. 37(11): 1595–1598 (Figure 1).

## General notes and troubleshooting

**General notes**

1. Pupil size is sensitive to light and noise; thus, it is important to ensure that the experiment room is quiet, and its illumination intensity remains constant throughout the whole procedure.

2. Pupil size changes primarily due to differences in physical brightness and contrast of stimuli; thus, it is crucial to match the materials used in different experimental conditions to facilitate better comparisons.

**Troubleshooting**

Problem 1: The participant is constantly blinking.

Possible cause: The participant is tired/not focused.

Solution: Pause the experiment and ask participants to take a break. Encourage them to rest their eyes and regain focus before continuing. Additionally, ensure the experimental environment is comfortable and free from distractions to minimize fatigue. If the issue persists, consider shortening the task duration or dividing it into smaller segments with breaks in between.

Problem 2: Fixation is not placed at the center of the screen.

Possible cause: The participant may have accidentally moved their head, disrupting the alignment of the eye-tracking system.

Solution: Administer a recalibration procedure to ensure accurate tracking. Guide the participant to reposition their head in the correct posture and repeat the calibration process. To prevent future occurrences, remind the participant to remain as still as possible during the experiment.

Problem 3: A bright area overlaps with the pupil area, making the eye tracker unable to track the participant’s eye.

Possible cause 1: The participant may be wearing eyeglasses that cause reflections from the screen light, creating bright areas that interfere with pupil detection.

Solution 1: If possible, have the participant switch to non-reflective lenses. Modify the lighting condition of the room to reduce glare on the glasses. Adjust the angle or position of the participant’s head to reduce reflections from the light source and recalibrate.

Possible cause 2: The parameters (e.g., contrast, brightness, or sensitivity) of the eye tracker may not be set properly, leading to difficulty in distinguishing the pupil from bright areas.

Solution 2: Modify the contrast and brightness settings of the eye tracker to better distinguish the pupil from surrounding bright areas. Recalibrate the eye tracker to ensure it is properly aligned with the participant’s eyes.

## References

[1] Grujic N., Polania R. and Burdakov D. (2024). Neurobehavioral meaning of pupil size. Neuron. 112(20): 3381 3395 3395. 10.1016/j.neuron.2024.05.029 38925124

[2] Lloyd B., de Voogd L. D., Mäki-Marttunen V. and Nieuwenhuis S. (2023). Pupil size reflects activation of subcortical ascending arousal system nuclei during rest. elife. 12: e84822. https://doi.org/10.7554/elife.84822 PMC10299825 37367220

[3] Joshi S. and Gold J. I. (2020). Pupil Size as a Window on Neural Substrates of Cognition. Trends Cognit Sci. 24(6): 466 480 480. 10.1016/j.tics.2020.03.005 32331857 PMC7271902

[4] de Gee J. W., Knapen T. and Donner T. H. (2014). Decision-related pupil dilation reflects upcoming choice and individual bias. Proc Natl Acad Sci USA. 111(5): E618–E625. https://doi.org/10.1073/pnas.1317557111 PMC3918830 24449874

[5] Graves J. E., Egré P., Pressnitzer D. and de Gardelle V. (2021). An implicit representation of stimulus ambiguity in pupil size. Proc Natl Acad Sci USA. 118(48): e2107997118. https://doi.org/10.1073/pnas.2107997118 PMC8640749 34819369

[6] Kloosterman N. A., Meindertsma T., van Loon A. M., Lamme V. A. F., Bonneh Y. S. and Donner T. H. (2015). Pupil size tracks perceptual content and surprise. Eur J Neurosci. 41(8): 1068 1078 1078. 10.1111/ejn.12859 25754528

[7] Oliva M. and Anikin A. (2018). Pupil dilation reflects the time course of emotion recognition in human vocalizations. Sci Rep. 8(1): 4871 10.1038/s41598-018-23265-x 29559673 PMC5861097

[8] Geangu E. and Vuong Q. C. (2023). Seven‐months‐old infants show increased arousal to static emotion body expressions: Evidence from pupil dilation. Infancy. 28(4): 820 835 835. 10.1111/infa.12535 36917082

[9] Hepach R. and Westermann G. (2016). Pupillometry in Infancy Research. J Cogn Dev. 17(3): 359 377 377. 10.1080/15248372.2015.1135801

[10] de Vries L., Fouquaet I., Boets B., Naulaers G. and Steyaert J. (2021). Autism spectrum disorder and pupillometry: A systematic review and meta-analysis. Neurosci Biobehav R. 120: 479 508 508. 10.1016/j.neubiorev.2020.09.032 33172600

[11] Yuan T., Wang L. and Jiang Y. (2024). Multi-level processing of emotions in life motion signals revealed through pupil responses. eLife. 12: e89873. https://doi.org/10.7554/elife.89873 PMC11473101 39401063

[12] Varazzani C., San-Galli A., Gilardeau S. and Bouret S. (2015). Noradrenaline and Dopamine Neurons in the Reward/Effort Trade-Off: A Direct Electrophysiological Comparison in Behaving Monkeys. J Neurosci. 35(20): 7866 7877 7877. 10.1523/jneurosci.0454-15.2015 25995472 PMC6795183

[13] Ren W., Huang K., Li Y., Yang Q., Wang L., Guo K., Wei P. and Zhang Y. Q. (2023). Altered pupil responses to social and non-social stimuli in Shank3 mutant dogs. Mol Psychiatry. 28(9): 3751 3759 3759. 10.1038/s41380-023-02277-8 37848709

[14] Schirmer A. and Adolphs R. (2017). Emotion Perception from Face, Voice, and Touch: Comparisons and Convergence. Trends Cognit Sci. 21(3): 216 228 228. 10.1016/j.tics.2017.01.001 28173998 PMC5334135

[15] Frith C. (2009). Role of facial expressions in social interactions. Philos Trans R Soc Lond B Biol Sci. 364(1535): 3453 3458 3458. 10.1098/rstb.2009.0142 19884140 PMC2781887

[16] Johansson G. (1973). Visual perception of biological motion and a model for its analysis. Percept Psychophys. 14(2): 201 211 211. 10.3758/bf03212378

[17] Troje N. F. (2008). 12 Retrieving Information from Human Movement Patterns. In: Shipley, T. F. and Zacks, J. M.(eds.). Understanding events: From perception to action. 308–334. 10.1093/acprof :oso/9780195188370.003.0014

[18] de Gelder B. (2006). Towards the neurobiology of emotional body language. Nat Rev Neurosci. 7(3): 242 249 249. 10.1038/nrn1872 16495945

[19] Actis-Grosso R., Bossi F. and Ricciardelli P. (2015). Emotion recognition through static faces and moving bodies: a comparison between typically developed adults and individuals with high level of autistic traits. Front Psychol. 6: e01570. 10.3389/fpsyg.2015.01570 PMC4615932 26557101

[20] Giberson T. R., Resick C. J. and Dickson M. W. (2005). Embedding Leader Characteristics: An Examination of Homogeneity of Personality and Values in Organizations. J Appl Psychol. 90(5): 1002 1010 1010. 10.1037/0021-9010.90.5.1002 16162072

[21] Spencer J. M. Y., Sekuler A. B., Bennett P. J., Giese M. A. and Pilz K. S. (2016). Effects of aging on identifying emotions conveyed by point-light walkers. Psychol Aging. 31(1): 126 138 138. 10.1037/a0040009 26765748

[22] Critchley H. D., Mathias C. J. and Dolan R. J. (2001). Neuroanatomical basis for first- and second-order representations of bodily states. Nat Neurosci. 4(2): 207 212 212. 10.1038/84048 11175883

[23] Lange J. and Lappe M. (2006). A Model of Biological Motion Perception from Configural Form Cues. J Neurosci. 26(11): 2894 2906 2906. 10.1523/jneurosci.4915-05.2006 16540566 PMC6673973

[24] Okon-Singer H., Lichtenstein-Vidne L. and Cohen N. (2013). Dynamic modulation of emotional processing. Biol Psychol. 92(3): 480 491 491. 10.1016/j.biopsycho.2012.05.010 22676964

[25] Shafer A. T., Matveychuk D., Penney T., O'Hare A. J., Stokes J. and Dolcos F. (2012). Processing of Emotional Distraction Is Both Automatic and Modulated by Attention: Evidence from an Event-related fMRI Investigation. J Cognit Neurosci. 24(5): 1233 1252 1252. 10.1162/jocn_a_00206 22332805 PMC4491634

[26] Kana R. K., Maximo J. O., Williams D. L., Keller T. A., Schipul S. E., Cherkassky V. L., Minshew N. J. and Just M. A. (2015). Aberrant functioning of the theory-of-mind network in children and adolescents with autism. Mol Autism. 6(1): 1 12 12. 10.1186/s13229-015-0052-x 26512314 PMC4624365

[27] Keifer C. M., Mikami A. Y., Morris J. P., Libsack E. J. and Lerner M. D. (2020). Prediction of social behavior in autism spectrum disorders: Explicit versus implicit social cognition. Autism. 24(7): 1758 1772 1772. 10.1177/1362361320922058 32484000 PMC7541482

[28] Kovarski K., Mennella R., Wong S. M., Dunkley B. T., Taylor M. J. and Batty M. (2019). Enhanced Early Visual Responses During Implicit Emotional Faces Processing in Autism Spectrum Disorder. J Autism Dev Disord. 49(3): 871 886 886. 10.1007/s10803-018-3787-3 30374763

[29] Burley D. T., Gray N. S. and Snowden R. J. (2017). As Far as the Eye Can See: Relationship between Psychopathic Traits and Pupil Response to Affective Stimuli. PLoS One. 12(1): e0167436. 10.1371/journal.pone.0167436 PMC5261620 28118366

[30] Baron-Cohen S., Wheelwright S., Skinner R., Martin J. and Clubley E. (2001). The autism-spectrum quotient(AQ): Evidence from asperger syndrome/high-functioning autism, males and females, scientists and mathematicians. J Autism Dev Disord. 31: 5 17 17. 10.1023/A:1005653411471 11439754

[31] Brainard D. H. (1997). The Psychophysics Toolbox. Spat Vis. 10(4): 433 436 436. 10.1163/156856897x00357 9176952

[32] Pelli D. G. (1997). The VideoToolbox software for visual psychophysics: transforming numbers into movies. Spat Vis. 10(4): 437 442 442. 10.1163/156856897x00366 9176953

[33] Groppe D. M., Urbach T. P. and Kutas M. (2011). Mass univariate analysis of event‐related brain potentials/fields I: A critical tutorial review. Psychophysiology. 48(12): 1711 1725 1725. 10.1111/j.1469-8986.2011.01273.x 21895683 PMC4060794

[34] Jamovi(2024). The jamovi project.

[35] Erdfelder E., Faul F. and Buchner A. (1996). GPOWER: A general power analysis program. Behav Res Methods Instruments Computers. 28(1): 1 11 11. 10.3758/bf03203630

[36] Kret M. E. and Sjak-Shie E. E. (2019). Preprocessing pupil size data: Guidelines and code. Behav Res Methods. 51(3): 1336 1342 1342. 10.3758/s13428-018-1075-y 29992408 PMC6538573

[37] Engbert R. and Kliegl R. (2003). Binocular Coordination in Microsaccades. The Mind's Eye. 103–117. 10.1016/b978-044451020-4/50007-4

[38] Troje N. F. (2002). Decomposing biological motion: A framework for analysis and synthesis of human gait patterns. J Vis. 2(5): 371 387 387. 10.1167/2.5.2 12678652

[39] Roether C. L., Omlor L., Christensen A. and Giese M. A. (2009). Critical features for the perception of emotion from gait. J Vis. 9(6): 15 15 15. 10.1167/9.6.15 19761306

[40] Chang D. H. F. and Troje N. F. (2009). Acceleration carries the local inversion effect in biological motion perception. J Vis. 9(1): 19 19 19. 10.1167/9.1.19 19271889

[41] Troje N. F. and Westhoff C. (2006). The Inversion Effect in Biological Motion Perception: Evidence for a“Life Detector”? Curr Biol. 16(8): 821 824 824. 10.1016/j.cub.2006.03.022 16631591

[42] Steinhauer S. R., Bradley M. M., Siegle G. J., Roecklein K. A. and Dix A. (2022). Publication guidelines and recommendations for pupillary measurement in psychophysiological studies. Psychophysiology. 59(4): e14035. https://doi.org/10.1111/psyp.14035 PMC9272460 35318693

[43] Einhäuser W., Stout J., Koch C. and Carter O. (2008). Pupil dilation reflects perceptual selection and predicts subsequent stability in perceptual rivalry. Proc Natl Acad Sci USA. 105(5): 1704 1709 1709. 10.1073/pnas.0707727105 18250340 PMC2234208

[44] Cheng Y., Yuan X. and Jiang Y. (2023). Eye pupil signals life motion perception. Atten Percept Psycho. 86(2): 579 586 586. 10.3758/s13414-023-02729-x 37258891

[45] Cheng Y., Liu W., Yuan X. and Jiang Y. (2021). The Eyes Have It: Perception of Social Interaction Unfolds Through Pupil Dilation. Neurosci Bull. 37(11): 1595 1598 1598. 10.1007/s12264-021-00739-z 34212296 PMC8566692

